# Targeting the Gut Microbiota-Bile Acid-FXR Axis: The Therapeutic Mechanism of Yudantong Decoction in Cholestatic Liver Disease

**DOI:** 10.1007/s11596-026-00191-y

**Published:** 2026-04-29

**Authors:** Xiao-ming Wu, Lin-yi Hou, Chang Liu, Yan Hu, Qiang He

**Affiliations:** Department of Traditional Chinese Medicine, Beijing Children’s Hospital, National Center for Children’s Health, Capital Medical University, Beijing, 100045 China

**Keywords:** Yudantong decoction, Cholestatic liver disease, Gut microbiota, Bile acids, Intestinal farnesoid X receptor, Bile salt hydrolase, NLRP3 inflammation, Intestinal Barrier, Fecal microbiota trasplantation

## Abstract

**Objective:**

To elucidate the therapeutic mechanism of Yudantong decoction (YDTD) in cholestatic liver disease (CLD), focusing on the gut microbiota-bile acid-intestinal farnesoid X receptor (FXR) axis.

**Methods:**

A CLD mouse model induced by α-naphthylisothiocyanate was treated with YDTD. Hepatic injury, gut microbiota composition (16S rRNA sequencing), bile acid profiles (high-performance liquid chromatography–tandem mass spectrometry, HPLC-MS/MS), intestinal FXR/NLRP3 signaling, and barrier function were assessed. Fecal microbiota transplantation, bile salt hydrolase (BSH) inhibition, and FXR antagonism were employed for mechanistic validation.

**Results:**

CLD mice exhibited hepatocellular steatosis, lobular necrosis, and elevated serum markers. These pathological changes were associated with gut dysbiosis, impaired bile acid metabolism via bile salt hydrolase (BSH) suppression, FXR signaling inhibition, and NLRP3 inflammasome activation. YDTD restored BSH activity and bile acid homeostasis, upregulated FXR expression, suppressed NLRP3 inflammasome activation, and improved intestinal barrier integrity. Fecal microbiota transplantation experiments confirmed that YDTD-modified microbiota mediated these therapeutic benefits, whereas pharmacological inhibition of BSH or FXR attenuated YDTD’s therapeutic effects.

**Conclusion:**

YDTD alleviates CLD, at least in part, by targeting the gut microbiota-bile acid-FXR signaling pathway, highlighting the gut microbiota as a promising therapeutic target for CLD.

## Introduction

Cholestatic liver disease (CLD) is a clinical syndrome caused by factors that impair bile formation, secretion, or excretion, leading to the accumulation of bile acids in the liver. It is a major cause of hospital visits and admissions related to pediatric liver disease [1]. If left untreated, chronic cholestasis can worsen hepatocellular injury, progress to liver fibrosis and cirrhosis, and pose a serious threat to children’s health and survival, ultimately contributing to pediatric mortality and disability [2]. Currently, therapeutic options for pediatric CLD remain limited. Ursodeoxycholic acid (UDCA) is commonly used but provides limited improvements in survival and histological outcomes, with approximately one-third of patients being unresponsive to treatment [3]. Yudantong decoction (YDTD), a traditional Chinese herbal formula, has been used for more than 50 years at Beijing Children’s Hospital to treat CLD owing to its hepatoprotective effects and favorable safety profile [4]. Clinical studies have demonstrated that YDTD reduces serum bilirubin levels and lowers the risk of cirrhosis in pediatric patients with CLD. However, the mechanism by which YDTD alleviates jaundice and preserves liver function remains poorly understood.

Recent studies have highlighted the central role of the gut microbiota–bile acid–intestinal farnesoid X receptor (FXR) axis in the pathogenesis of CLD. In CLD, the gut microbial ecosystem is disrupted, characterized by a decrease in beneficial bacteria (e.g., *Bifidobacterium* and *Lactobacillus*) and an increase in pathogenic species (e.g., *Escherichia coli* and *Enterococcus*) [5]. The gut microbiota and its metabolites greatly influence bile acid metabolism, which is crucial in CLD progression [6]. Gut microbial enzymes, such as bile salt hydrolases (BSHs), facilitate the conversion of primary bile acids into unconjugated bile acids, which can subsequently be converted into secondary bile acids. This process primarily occurs in the small intestine, with Lactobacillus and Bifidobacterium being key sources of BSH activity [7]. Primary bile acids, including cholic acid (CA) and chenodeoxycholic acid (CDCA), are synthesized in the liver, conjugated with taurine or glycine, and secreted into the small intestine. Conjugated bile acids act as FXR antagonists and are inefficiently excreted in feces [8]. By contrast, unconjugated and secondary bile acids are more readily excreted and act as FXR activators, contributing to the maintenance of intestinal barrier integrity [9]. The abnormal activation of the nucleotide-binding oligomerization domain-like receptor protein 3 (NLRP3) inflammasome is closely related to intestinal barrier dysfunction in CLD. As a key component of innate immunity, the NLRP3 inflammasome is activated by pathogen-associated molecular patterns (PAMPs) and danger-associated molecular patterns (DAMPs) via pattern recognition receptors (PRRs). The NLRP3 inflammasome is a multi-protein complex composed of NLRP3, apoptosis-associated speck-like protein containing a CARD (ASC), and pro-caspase-1. Upon assembly and activation, pro-caspase-1 is cleaved into active caspase-1, which subsequently processes the precursors of IL-1β and IL-18 into their mature forms, promoting inflammatory cascade reactions [10]. Upon activation, FXR interacts with NLRP3 and caspase-1 to suppress inflammasome activation, exert anti-inflammatory effects, and stabilize the intestinal barrier [10, 11]. In children with CLD, a reduction in BSH-producing bacteria leads to elevated levels of conjugated bile acids in the ileum, impairing bile acid excretion and suppressing intestinal FXR signaling [12]. This disruption increases intestinal permeability, allowing pathogens to bypass the intestinal barrier, enter the liver via the portal vein, and trigger liver inflammation and fibrosis [13].

To investigate the mechanism underlying the therapeutic effects of YDTD in CLD, in this study, we employed 16S rRNA gene sequencing, high-performance liquid chromatography-tandem mass spectrometry (HPLC–MS/MS), immunohistochemistry, and immunofluorescence to evaluate the effects of YDTD on gut microbiota composition, bile acid metabolism, intestinal FXR expression, and barrier function in cholestatic mice. Furthermore, through microbiota transplantation, BSH inhibition, and FXR-specific blockade, we explored the key mechanisms involved in YDTD-mediated regulation of the microbiota-bile acid-FXR axis in alleviating cholestasis and protecting liver function. Our results demonstrated that YDTD reshapes the gut microbiota, enhances bile acid metabolism, activates intestinal FXR signaling, inhibits NLRP3 inflammasome activation, stabilizes the intestinal barrier, and ultimately ameliorates cholestasis and improves liver function.

## Materials and Methods

### Preparation of YDTD

The herbal formula for YDTD comprises 13 natural medicinal components: *Artemisiae Scopariae Herba* (Yinchen), *Lysimachiae Herba* (Jinqiancao), *Phellodendri Chinensis Cortex* (Huangbai), *Tetrapanacis Medulla* (Tongcao), *Indigo Naturalis* (Qingdai), *Salviae Miltiorrhizae Radix Et Rhizoma* (Danshen), *Lycopi Herba* (Zelan), *Hordei Fructus Germinatus* (Maiya), *Poria* (Fuling), *Atractylodis Macrocephalae Rhizoma* (Baizhu), *Draconis Sanguis* (Xuejie), *Alumen* (Baifan), and *Succinum* (Hupo) [14]. YDTD was obtained from Beijing Children’s Hospital (China) and subsequently diluted to a final volume of 200 mL, yielding a crude drug concentration of 0.366 g/mL.

### Animals and Interventions

**Fig. 1:**
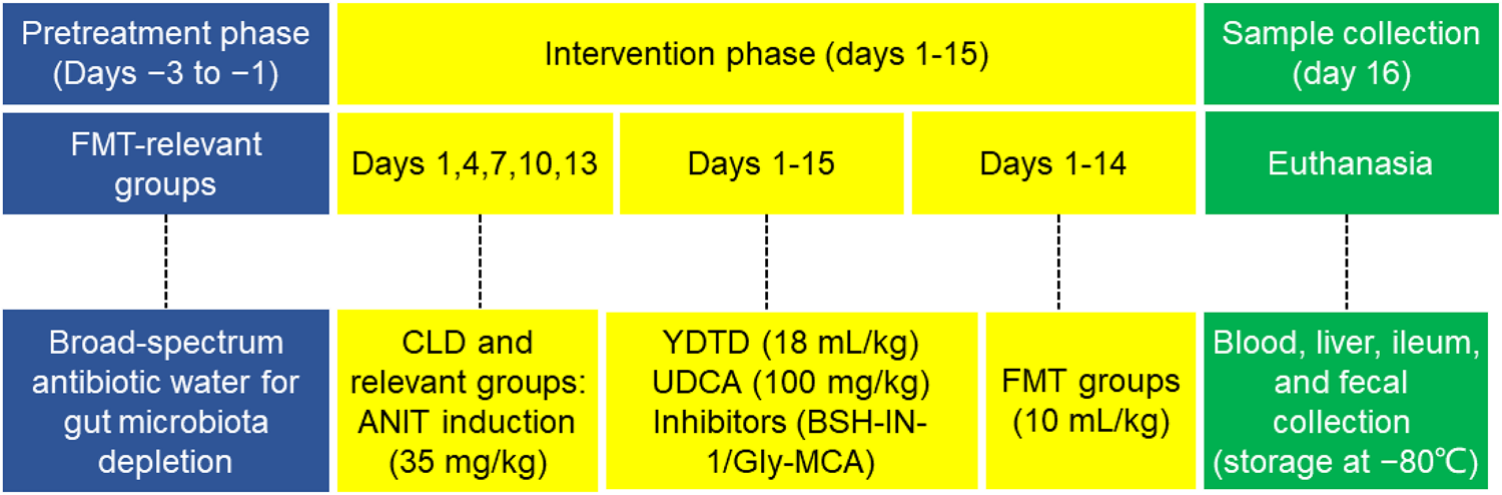
Experimental design and timeline

A total of 90 male C57BL/6 mice (8–10 weeks old; weighing 20–22 g, mean 21.1 ± 0.9 g) were randomly assigned to nine groups, with ten mice per group: (1) vehicle, (2) CLD, (3) YDTD, (4) UDCA, (5) YDTD + UDCA, (6) YDTD + FMT^CLD^, (7) CLD + FMT^YDTD^, (8) YDTD + BSH-IN-1, and (9) YDTD + Gly-MCA groups (FMT: fecal microbiota transplant; BSH-IN-1: BSH inhibitor; Gly-MCA: FXR inhibitor). Based on prior studies [15] and preliminary experiments, mice in groups 2–9 were administered α-naphthylisothiocyanate (ANIT; MedChemExpress, HY-W540630, USA; 35 mg/kg, oral gavage) on days 1, 4, 7, 10, and 13, whereas those in the vehicle group received salad oil. From day 1 to 15, mice in groups 3, 5, 6, 8, and 9 received daily oral gavage of YDTD (18 mL/kg), while those in groups 4 and 5 received daily oral gavage of UDCA (Ursofalk, Dr. Falk Pharma GmbH, Germany; 100 mg/kg, suspended in water at 5 mg/mL); the vehicle (1) and CLD (2, 7) groups received an equivalent volume of distilled water. For FMT groups (6, 7), donor fecal samples from matched mice were processed into a bacterial suspension, and recipient mice underwent gut microbiota depletion with broad-spectrum antibiotics (0.5 g/L vancomycin, 1 g/L neomycin sulfate, 1 g/L metronidazole, and 1 g/L ampicillin) in drinking water for 3 days [16] (FMT^CLD^: microbiota from CLD mice; FMT^YDTD^: microbiota from YDTD mice). Subsequently, mice were administered a bacterial suspension (10 mL/kg) by oral gavage daily for 14 days. BSH-IN-1 (MedChemExpress, HY-135659; 10 mg/kg/day) and Gly-β-MCA (MedChemExpress, HY-114392; 10 mg/kg/day) were administered orally for 14 days in groups 8 and 9. On day 16, mice were euthanized. Blood was collected from the inferior vena cava, and the liver, ileum, and fecal samples were harvested and stored at −80 °C until further analysis (Fig. 1).

### Assessment of Hepatic Function

Blood samples were allowed to clot at room temperature for 3 h, then centrifuged at 3,000 rpm for 10 min. The serum was subsequently collected, and the levels of alanine aminotransferase (ALT), aspartate aminotransferase (AST), gamma-glutamyl transferase (GGT), alkaline phosphatase (ALP), total bilirubin (TBil), direct bilirubin (DBil), and total bile acid (TBA) in the mouse serum were analyzed using a Mindray BS-600 M automated biochemical analyzer (Shenzhen Mindray Bio-Medical Electronics Co., Ltd., China).

### Histological Examination of Liver Tissue Using Hematoxylin and Eosin (H&E) and Sirius Red Staining

Liver tissues were fixed in 4% formalin at room temperature, embedded in paraffin at 60–62 °C, and sectioned into 4-µm-thick slices. The sections were deparaffinized in xylene and rehydrated through a graded ethanol series. For H&E staining, sections were stained with hematoxylin (Beyotime, C0107, China) for 3 min, rinsed with tap water, and counterstained with eosin (Beyotime, C0109) for 1 min. Following dehydration and clearing with xylene, sections were sealed with neutral resin and examined under a light microscope (Olympus Corporation, BX53, Japan) at 200× magnification.

For Sirius Red staining, sections were deparaffinized in xylene, rehydrated through graded ethanol, and rinsed with distilled water. After hematoxylin staining for 5–10 min, excess stain was removed, and sections were washed thoroughly. Sections were then stained with Sirius Red (Beyotime, Y026187) for 15–30 min, rinsed, dehydrated in ethanol, clarified with xylene, and sealed with neutral gum. Under microscopic observation (Olympus Corporation, BX53), collagen fibers appeared red, while nuclei ranged from brown to black.

### Immunohistochemistry

Tissue sections were deparaffinized, hydrated, and incubated with a peroxidase-blocking reagent for 5 min. After rinsing with distilled water and washing twice with phosphate-buffered saline (PBS; Beyotime, C0221A), antigen retrieval was performed with sodium citrate buffer, followed by additional PBS washes. Sections were blocked with 5% goat serum for 10–30 min and incubated with diluted primary antibodies overnight at 4 °C. The following primary antibodies were used: rabbit anti-α-SMA monoclonal antibody (Abcam, ab124964, 1:1000 dilution, UK); rabbit anti-CK19 monoclonal antibody (Abcam, ab52625, 1:500 dilution); rabbit anti-FXR1 polyclonal antibody (Abcam, ab155124, 1:100 dilution); rabbit anti-NLRP3 polyclonal antibody (Proteintech, 30109-1-AP, 1:100 dilution, USA); rabbit anti-caspase-1 polyclonal antibody (Abcam, ab138483, 1:200 dilution); and rabbit anti-IL-1β polyclonal antibody (Proteintech, 26048-1-AP, 1:200 dilution). After five PBS washes, sections were incubated with diluted horseradish peroxidase (HRP)-conjugated goat anti-rabbit IgG secondary antibody (Beyotime, A0208, 1:200 dilution) for 30 min at 37 °C, followed by five additional PBS washes. The sections were treated with DAB working solution for 5–10 min, washed three times with PBS, and rinsed with distilled water. Counterstaining was subsequently performed with hematoxylin, followed by rinsing with distilled water and washing with PBS. After dehydration and clearing, the sections were sealed in neutral resin and observed under a light microscope (Olympus Corporation, BX53).

### Flow Cytometry

Fresh liver tissues were collected, minced into small pieces (approximately 1–2 mm^3^) using sterile scissors, and gently pressed through a 70-μm cell strainer to obtain single-cell suspensions for subsequent experiments. The cells were incubated with antibodies in the dark at 37 °C for 30 min. After incubation, cells were centrifuged at 300 × *g* for 5 min at 4 °C to remove the supernatant and washed three times with PBS. The precipitate was resuspended in 200 µL of PBS and analyzed using flow cytometry (BD Biosciences, FACSCanto II, USA).

### Tissue Immunofluorescence Staining

Tissue sections were deparaffinized, rehydrated, and subjected to antigen retrieval using microwave heating for 10–15 min. After blocking nonspecific binding with sheep serum (Beyotime, C0265) for 60 min at 37 °C, sections were incubated with primary antibodies overnight at 4 °C. The following primary antibodies were used: mouse anti-E-Cadherin monoclonal antibody (Abcam, ab231303, 1:100 dilution); rabbit anti-Occludin monoclonal antibody (Abcam, ab216327, 1:100 dilution). The sections were washed three times with PBS and incubated with a fluorescein-labeled secondary antibody (goat anti-mouse IgG; Beyotime, A0521, 1:500 dilution) for 60 min at 37 °C in the dark for E-Cadherin detection, and goat anti-rabbit IgG (Beyotime, A0516, 1:500 dilution) for occludin detection. After additional PBS washes, sections were sealed and stored in the dark at 4 °C. Observation and imaging were performed using a fluorescence microscope (Olympus Corporation, BX53).

### Western Blot Analysis

Total protein was extracted from liver tissues using a lysis buffer containing protease and phosphatase inhibitors, and the mixture was subsequently centrifuged at 15,000× *g* for 10 min at 4 °C. The supernatant was transferred to a new tube on ice, and protein concentration was determined using the bicinchoninic acid (BCA) method with a BCA protein assay kit (Beyotime, P0010). Equal amounts of protein were separated by sodium dodecyl sulfate (SDS)–polyacrylamide gel electrophoresis and transferred onto a polyvinylidene fluoride membrane (Beyotime, FFP24). The membrane was blocked overnight at 4 °C, then incubated with primary antibodies in blocking buffer. The following primary antibodies were used: rabbit anti-NLRP3 monoclonal antibody (Abcam, ab263899, 1:1000 dilution); rabbit anti-caspase-1 monoclonal antibody (Cell Signaling Technology, 83383, 1:1000 dilution, USA); rabbit anti-IL-1β polyclonal antibody (Proteintech, 26048–1-AP, 1:1000 dilution, USA); and mouse anti-GAPDH monoclonal antibody (Proteintech, 60004–1-Ig, 1:5000 dilution). After washing with Tris-buffered saline containing Tween-20, the membrane was incubated with secondary antibodies at room temperature for 1 h: goat anti-rabbit IgG (Beyotime, A0208, 1:1000 dilution) for detection of rabbit primary antibodies (NLRP3, caspase-1, IL-1β), and goat anti-mouse IgG (Beyotime, A0216, 1:1000 dilution) for detection of mouse primary antibody (GAPDH). Protein bands were visualized using a chemiluminescence analyzer (Tanon, 5200, China).

### Microbial 16S rDNA Sequencing and Information Analysis

Microbial 16S rDNA sequencing was performed by Beijing Novogene Biotech Co., Ltd (China). Genomic DNA was extracted using the SDS method, and its purity and concentration were assessed by agarose gel electrophoresis [17]. The extracted DNA was diluted to 1 ng/μL and used as a template for polymerase chain reaction (PCR) amplification with specific barcode-containing primers. The PCR products were analyzed by 2% agarose gel electrophoresis, and the target bands were purified using a Qiagen gel extraction kit (QIAGEN, 28706, Germany). A library was prepared using the TruSeq™ DNA PCR-Free Kit (Illumina, FC-121–3001, USA), and quality was evaluated using Qubit fluorometry (Thermo Fisher Scientific, USA) and quantitative PCR (Applied Biosystems, 4368814, USA). Sequencing was performed on a NovaSeq 6000 instrument (Illumina, 20028312).

### Quantitative Analysis of Bile Acids

Bile acids were quantitatively analyzed using HPLC–MS/MS (API3200 Q-TRAP, SCIEX, USA). Liver, ileum, and fecal samples were homogenized and centrifuged, and 50 μL of the resulting supernatant was mixed with 150 μL of acetonitrile to precipitate proteins. After vortexing and centrifugation, the supernatant was collected for subsequent analysis. HPLC conditions included an MSLab HP-C18 column (150 × 4.6 mm, 5 μm), a column temperature of 50 °C, and a flow rate of 0.5 mL/min. The mobile phase consisted of acetonitrile (A) and water (B), both containing 5 mmol/L of ammonium acetate and 0.05‰ of formic acid. MS analysis was performed with negative electrospray ionization in negative ion mode, using multiple reaction monitoring.

### BSH Protein Level Measurement by Enzyme-Linked Immunosorbent Assay (ELISA)

BSH protein levels were measured using the Mouse BSH ELISA Kit (Catalog no. BY-EM222952, Nanjing Boyan Biotechnology, China) according to the manufacturer’s instructions. Samples were incubated in a 96-well plate coated with antibodies specific to BSH. After washing, a horseradish peroxidase–conjugated secondary antibody was added, followed by a substrate solution. The reaction was terminated, and optical density (OD) value was measured at 450 nm using a microplate reader (BioTek, EPOCH, USA). BSH concentrations were determined by comparing OD values to a standard curve of known recombinant BSH standards.

### Statistical Analysis

Data were analyzed using IBM SPSS Statistics, version 26. Measurement data are presented as means ± standard deviation (*x̅* ± *s*). One-way analysis of variance was used to compare multiple groups, with pairwise comparisons conducted using the least significant difference test, assuming homogeneity of variance. If the variance was not homogeneous, the Welch test was applied, followed by pairwise comparisons conducted using the Games–Howell test. Statistical significance was set at *P* < 0.05.

## Results

### YDTD Protects the Liver from Injury, Fibrosis, and Inflammation in Cholestasis

**Fig. 2:**
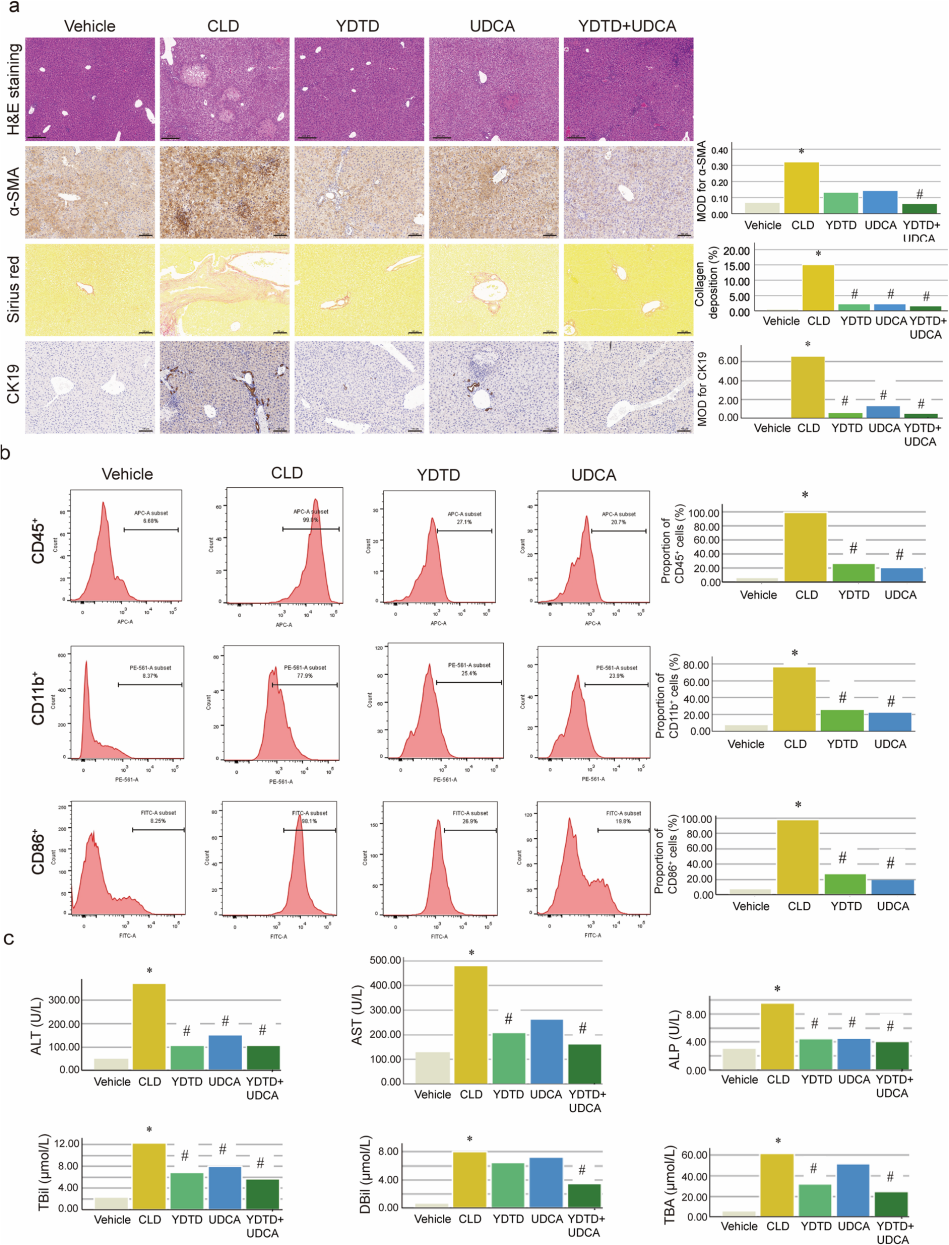
YDTD protects the liver from inflammation and fibrosis during cholestasis. **a** Representative H&E and Sirius Red staining of liver tissues from each mouse group (20× magnification; scale bars: 200 μm for H&E and 100 μm for Sirius Red). Immunohistochemical staining for α-SMA and CK19, and the protein expression was quantified by measuring the mean optical density (MOD) (20× magnification; scale bar: 100 μm). **b** Flow cytometry analysis of the proportions of CD11b^+^, CD86^+^, and CD45^+^ immune cells in the livers of each experimental group. **c** Comparison of liver function among the different groups (n = 4). ^*^P < 0.05 vs. the vehicle group, ^#^*P* < 0.05 vs. the CLD group

To investigate the protective effect of YDTD on ANIT-induced cholestatic liver injury, we treated mice with YDTD, with UDCA serving as a reference control. Histopathological analysis revealed severe liver damage in cholestatic mice, characterized by hepatocyte necrosis, cellular edema, and inflammatory infiltration. Treatment with either YDTD or UDCA significantly alleviated these pathological changes, as evidenced by improvements in H&E staining, reduced collagen deposition in Sirius Red staining, and decreased CK19-positive area. However, while both YDTD and UDCA individually led to a moderate reduction in α-SMA expression, a statistically significant downregulation was achieved only when they were administered in combination. (Fig. 2a).

Sirius Red staining and α-smooth muscle actin (α-SMA) expression showed increased collagen deposition and liver fibrosis in cholestatic mice. By contrast, treatment with YDTD and UDCA significantly reduced fibrosis, with the combined treatment exhibiting the greatest antifibrotic effect. Immunohistochemistry revealed reduced cytokeratin 19 (CK19) expression, indicating that both YDTD and UDCA suppressed cholangiocyte hyperplasia (Fig. 2a).

Flow cytometric analysis showed increased proportions of liver immune cells (CD11b^+^, CD86^+^, and CD45^+^) in CLD mice, which were significantly reduced after treatment with YDTD and UDCA (Fig. 2b).

Serum analyses revealed significantly elevated levels of ALT, AST, ALP, TBA, TBil, and DBil in CLD mice. Treatment with YDTD markedly reduced the levels of ALT, AST, ALP, TBA, and TBil, while combined treatment with YDTD and UDCA resulted in a greater overall improvement in liver function (Fig. 2c).

Collectively, these results indicate that both YDTD and UDCA effectively attenuate ANIT-induced cholestatic liver injury, with combined treatment providing enhanced hepatoprotective effects.

### YDTD Ameliorates Gut Microbiota and Increases BSH Protein Levels in CLD Mice

To investigate whether the therapeutic effects of YDTD on CLD are mediated by modulation of the gut microbiota, we performed 16S rRNA gene sequencing on fecal samples from vehicle, CLD, and YDTD-treated mice.

**Fig. 3:**
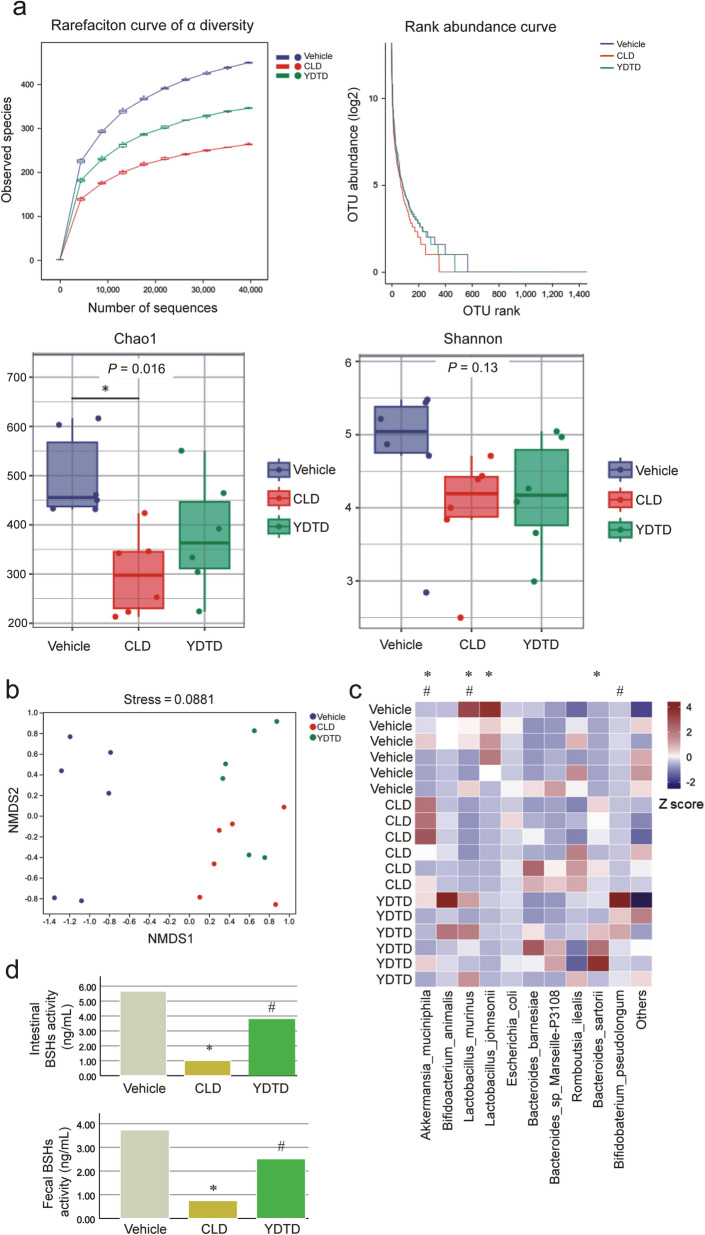
YDTD treatment modulates gut microbiota composition and restores intestinal BSH protein levels in CLD mice. **a** Rarefaction curves showing the sequencing depth, rank abundance curves, and comparison of α-diversity indices (Chao1 and Shannon) among the vehicle, CLD, and YDTD groups. **b** NMDS analysis. **c** Species-level analysis of microbial differences in the intestinal flora among the three experimental groups. **d** BSH protein levels in intestinal and fecal samples (n = 6). ^*^P < 0.05 vs. the vehicle group, ^#^*P* < 0.05 vs. the CLD group

α-diversity analysis showed that species richness was lower in cholestatic mice than in normal mice, with the YDTD group falling between the two groups. Chao1 index analysis revealed a significant difference between the vehicle and CLD groups (*P* = 0.016), whereas no significant difference was observed in Shannon diversity (Fig. 3a).

β-diversity analysis, through non-metric multidimensional scaling (NMDS) and analysis of similarities [18], revealed notable differences in microbial community composition between cholestatic and normal mice. YDTD treatment significantly altered the gut microbiota profile in CLD mice (Fig. 3b, Table 1).

**Table 1:** Analysis of similarities

Group 1	Group 2	*R* value	*P* value
Vehicle	CLD	0.779576	0.001
Vehicle	YDTD	0.516741	0.001
CLD	YDTD	0.293527	0.001

Taxonomic composition analysis at the genus and species levels identified specific microbial taxa altered by CLD and YDTD treatment. Compared with normal mice, the CLD group exhibited increased abundances of *Akkermansia muciniphila* and *Bacteroides sartorii*, along with reduced levels of *Lactobacillus johnsonii* and *Lactobacillus murinus*. YDTD treatment decreased the abundance of *A. muciniphila* and increased the relative abundances of *L. murinus* and *Bifidobacterium pseudolongum* in CLD mice (Fig. 3c).

As *Lactobacillus* and *Bifidobacterium* species produce BSH [19], we measured BSH protein levels in intestinal contents and fecal samples. BSH levels were significantly reduced in CLD mice but were markedly restored following YDTD treatment in both the intestinal and fecal samples (Fig. 3d).

Together, these findings indicate that YDTD promotes the enrichment of beneficial gut bacteria and restores BSH protein levels in CLD mice.

### YDTD Treatment Alters the Bile Acid Profile and Promotes Fecal Bile Acid Excretion in CLD Mice

**Fig. 4:**
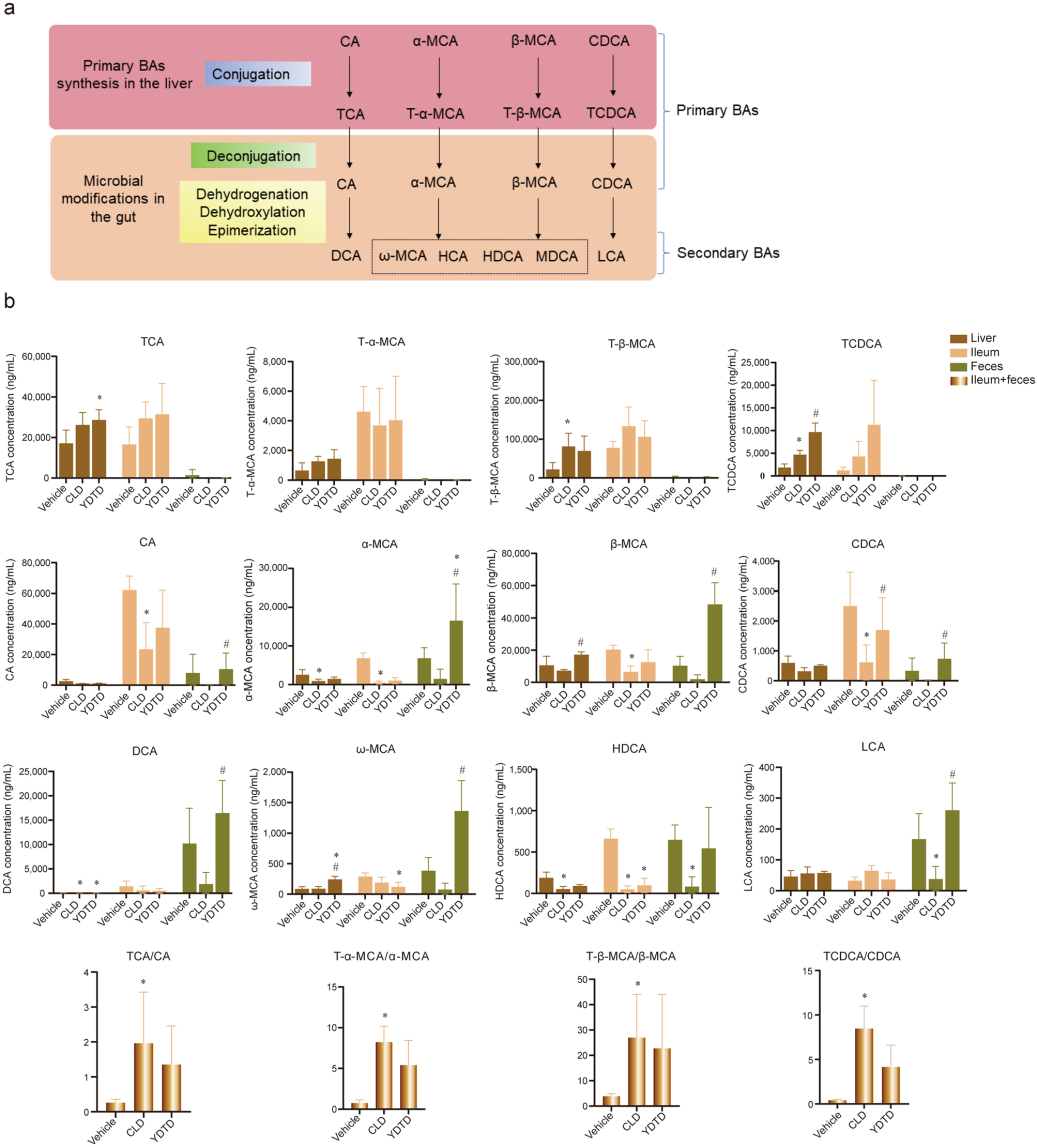
YDTD treatment alters the bile acid profile in cholestatic mice. **a** Schematic overview of bile acid metabolism in the enterohepatic circulation, showing the major primary bile acids (CA, MCAs, CDCA), their taurine-conjugated forms, and the generation of secondary bile acids (DCA, ω-MCA, HDCA, LCA) via gut microbial enzymes (BSH and 7α-dehydroxylase). **b** Quantitative analysis of bile acid levels in liver, ileum, and fecal samples from vehicle, CLD, and YDTD-treated mice, measured by HPLC–MS/MS (n = 4). ^*^P < 0.05 vs. the vehicle group, ^#^*P* < 0.05 vs. the CLD group

To investigate the effect of YDTD on bile acid metabolism in cholestasis, we quantified bile acid levels in liver, ileum, and fecal samples from vehicle, CLD, and YDTD-treated mice using HPLC–MS/MS. The major bile acid species analyzed included primary bile acids (CA, α-muricholic acid [α-MCA], β-MCA, CDCA), their taurine-conjugated forms (TCA, T-α-MCA, T-β-MCA, TCDCA), and secondary bile acids (deoxycholic acid [DCA], ω-MCA, and hyodeoxycholic acid [HDCA], lithocholic acid [LCA]) [20] (Fig. 4a).

In CLD mice, bile acid homeostasis was markedly disrupted, characterized by hepatic and ileal accumulation of conjugated bile acids (TCA, T-α-MCA, T-β-MCA, and TCDCA) and a significant reduction in unconjugated and secondary bile acids in the intestine and feces (Fig. 4b), a pattern consistent with impaired bile acid deconjugation and biotransformation in the gut. YDTD treatment significantly reversed these abnormalities, as YDTD-treated mice exhibited increased unconjugated bile acid levels (CA, α-MCA, β-MCA, and CDCA) in the ileum and feces, markedly elevated secondary bile acid levels (DCA, ω-MCA, HDCA, and LCA) in feces (Fig. 4b).

These changes indicate that YDTD promotes the deconjugation and 7α-dehydroxylation of primary bile acids, likely through its modulatory effects on gut microbiota composition and BSH activity, thereby facilitating bile acid excretion and alleviating hepatic bile acid accumulation in cholestatic mice.

### YDTD Alleviates Cholestasis-Induced Intestinal Barrier Injury by Modulating the Intestinal FXR/NLRP3 Signaling Axis

Different bile acid subtypes exert distinct regulatory effects on the FXR signaling pathway. Conjugated bile acids (e.g., TCA and TCDCA) inhibit FXR activation, whereas unconjugated bile acids (e.g., CA and CDCA) act as strong FXR agonists [21]. FXR activation negatively regulates the NLRP3 inflammasome, reducing intestinal inflammation and enhancing barrier function [22].

**Fig. 5:**
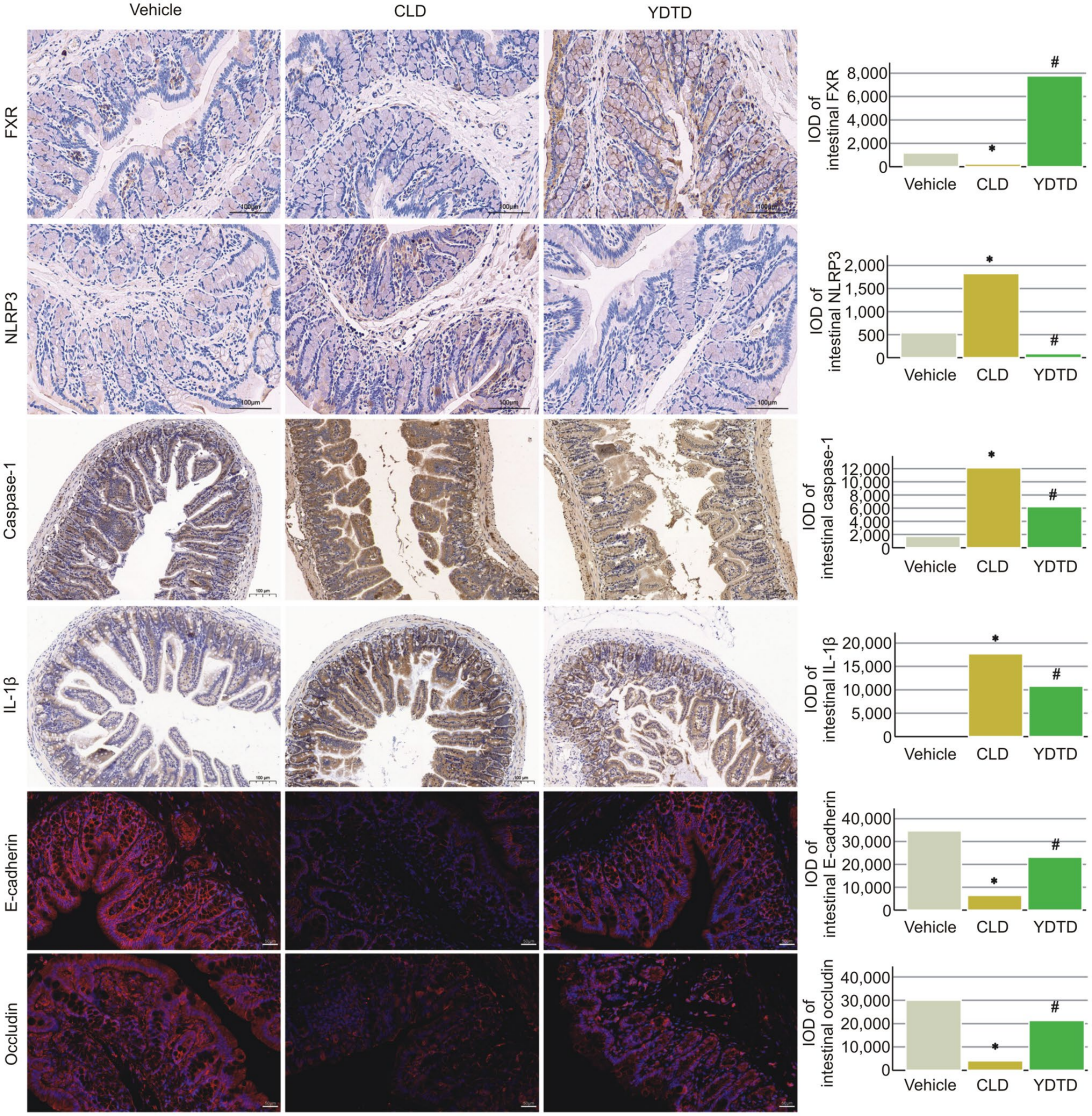
YDTD ameliorates cholestasis-induced intestinal injury by modulating the FXR/NLRP3 signaling pathway and restoring barrier integrity. Representative images show immunohistochemical staining for FXR, NLRP3 (200× magnification; scale bars: 50 μm), caspase-1, and IL-1β (20× magnification; scale bars: 100 μm), and immunofluorescence staining of tight junction proteins E-cadherin and occludin (200× magnification; scale bars: 50 μm) in mouse intestinal tissues. Quantification of the above results was performed by measuring the integrated optical density (IOD) (n = 3). ^*^P < 0.05 vs. the vehicle group, ^#^*P* < 0.05 vs. the CLD group

To explore the mechanisms underlying YDTD’s protective effects on the intestinal barrier in cholestatic mice, we examined the expression of FXR, NLRP3, and intestinal barrier markers. In cholestatic mice, FXR expression was significantly reduced, whereas the levels of NLRP3, caspase-1, and interleukin 1-beta (IL-1β) were markedly elevated. YDTD treatment upregulated FXR expression and downregulated the expression of inflammasome-related proteins. Immunofluorescence analysis also showed that YDTD increased the expression of tight junction proteins, such as E-cadherin and occludin, restoring the intestinal barrier (Fig. 5).

Collectively, these findings demonstrate that YDTD alleviates cholestasis-induced intestinal barrier injury by remodeling the bile acid profile, thereby reactivating intestinal FXR signaling and suppressing NLRP3 inflammasome activation.

### Reversal of YDTD-Mediated Protection in CLD Mice by Microbiota Transplantation, BSH Inhibition, and FXR Blockade

**Fig. 6:**
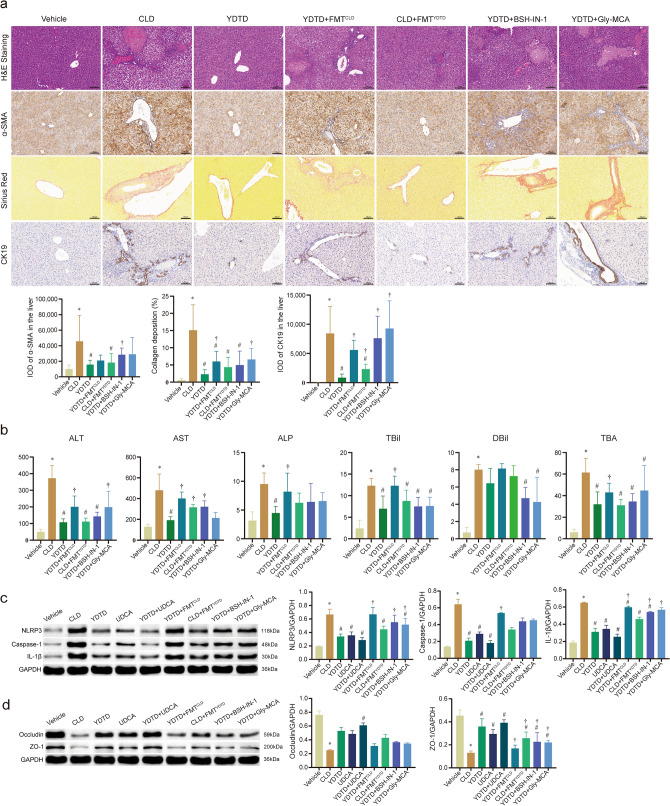
YDTD protects against cholestatic liver injury and intestinal barrier dysfunction via gut microbiota and FXR signaling. **a** Representative histological and immunohistochemical images and quantification analysis of liver sections (H&E, Sirius Red, α-SMA, and CK19 staining; 20× magnification; scale bar: 100 μm). **b** Serum biochemistry assessing liver function. **c, d** Western blot analysis of protein expression for NLRP3 inflammasome components (NLRP3, caspase-1, and IL-1β) in liver tissues (**c**) and tight junction proteins (occludin and ZO-1) (**d**) in intestinal tissues (n = 3). ^*^P < 0.05 vs. the vehicle group, ^#^*P* < 0.05 vs. the CLD group, ^†^*P* < 0.05 vs. the YDTD group

To systematically validate the crucial role of the gut microbiota-FXR axis in YDTD’s hepatoprotective effects, a series of functional intervention experiments was performed. The experimental design included three complementary approaches: (1) FMT from mice in the CLD group to those in the YDTD treatment group; (2) use of specific inhibitors to attenuate intestinal BSH activity in the YDTD group; and (3) pharmacological inhibition of intestinal FXR signaling in the YDTD group. As depicted in Fig. 6, all interventions significantly reduced the therapeutic effects of YDTD, resulting in increased liver inflammation, aggravated fibrosis, reactivation of bile duct hyperplasia, deterioration of liver function, and impairment of intestinal barrier function. Notably, transplantation of microbiota from YDTD-treated mice into cholestatic mice partially restored liver function and intestinal barrier integrity, highlighting the microbiota’s central role in mediating YDTD’s protective effects.

These findings demonstrate that the hepatoprotective and intestinal barrier–restorative effects of YDTD depend on the gut microbiota-BSH-FXR axis, where (1) microbiota composition serves as the initiating factor, (2) BSH-mediated bile acid metabolism functions as the main regulatory process, and (3) FXR activation acts as the downstream effector mechanism. These results support the multitarget action mechanism of YDTD.

### Reversal of YDTD-Mediated Bile Acid Metabolism in CLD Mice by Microbiota Transplantation and BSH Inhibition

**Fig. 7:**
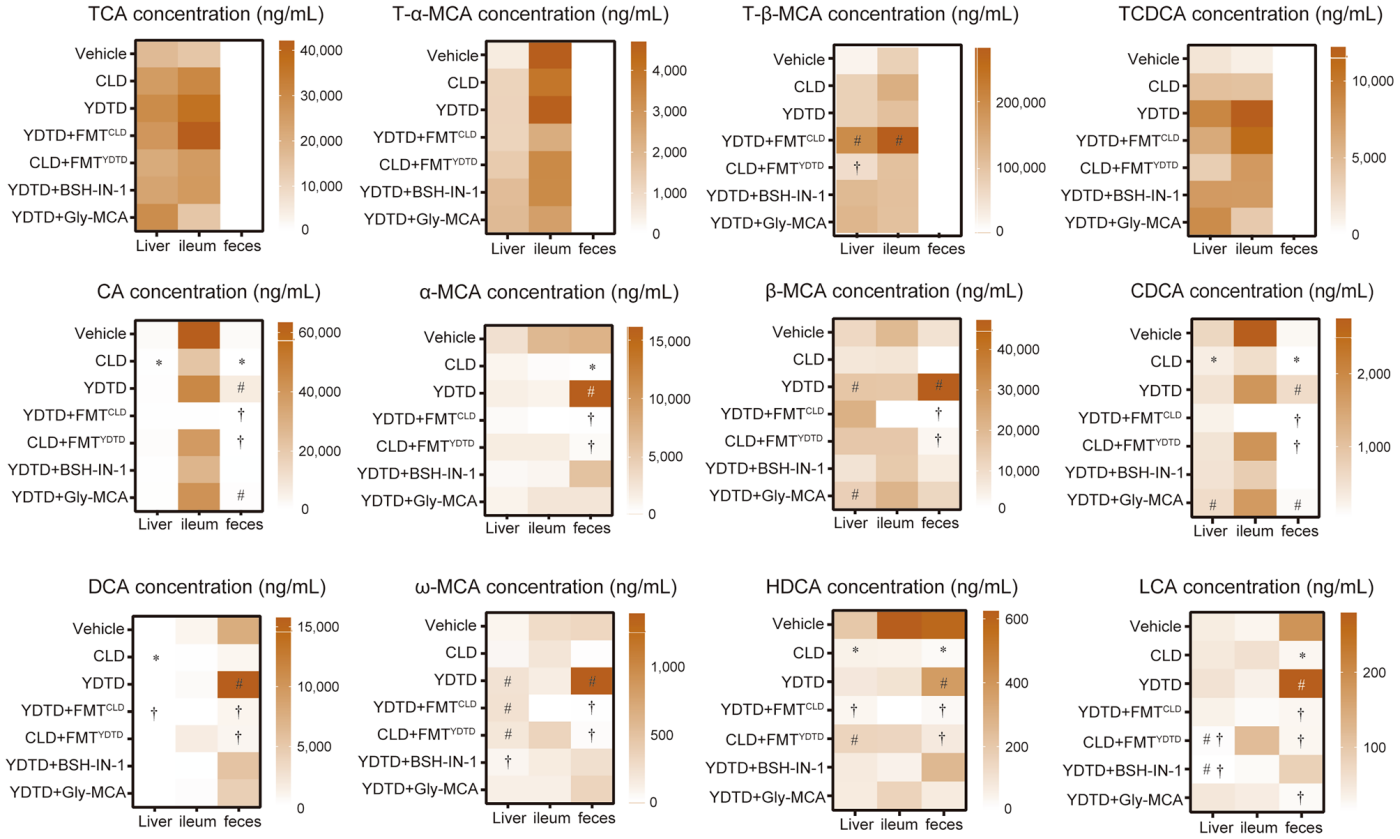
Quantitative analysis of bile acid composition in liver, ileum, and fecal samples from vehicle, CLD, YDTD, YDTD + FMT^CLD^ (fecal microbiota transplant from CLD mice), CLD + FMT^YDTD^ (fecal microbiota transplant from YDTD-treated mice), YDTD + BSH-IN-1 (BSH inhibitor), and YDTD + Gly-MCA (FXR antagonist) groups (n = 4). ^*^P < 0.05 vs. the vehicle group, ^#^*P* < 0.05 vs. the CLD group, ^†^*P* < 0.05 vs. the YDTD group

To further elucidate the roles of intestinal microbiota and BSH activity in YDTD-mediated bile acid metabolism, we compared bile acid profiles across seven experimental groups: vehicle, CLD, YDTD, YDTD + FMT^CLD^, CLD + FMT^YDTD^, YDTD + BSH-IN-1, and YDTD + Gly-MCA. As shown in Fig. 7, fecal microbiota transplantation from CLD mice into YDTD-treated mice (YDTD + FMT^CLD^) significantly reduced the levels of unconjugated and secondary bile acids—including CA, α-MCA, β-MCA, CDCA, DCA, ω-MCA, HDCA, and LCA—compared to the YDTD group. A similar reduction was observed following pharmacological inhibition of BSH activity (YDTD + BSH-IN-1). Conversely, transplantation of microbiota from YDTD-treated mice into cholestatic mice (CLD + FMT^YDTD^) significantly increased the levels of unconjugated and secondary bile acids compared to the CLD group. Notably, FXR blockade with Gly-MCA (YDTD + Gly-MCA) did not alter the bile acid profile, confirming that FXR acts downstream of bile acid metabolism rather than regulating it directly. (Fig. 7).

These findings demonstrate that both the gut microbiota composition and BSH activity are essential for YDTD-mediated bile acid metabolism. The ability of YDTD-shaped microbiota to improve bile acid metabolism in cholestatic mice further supports the causal role of the microbiota in YDTD’s therapeutic mechanism.

**Fig. 8:**
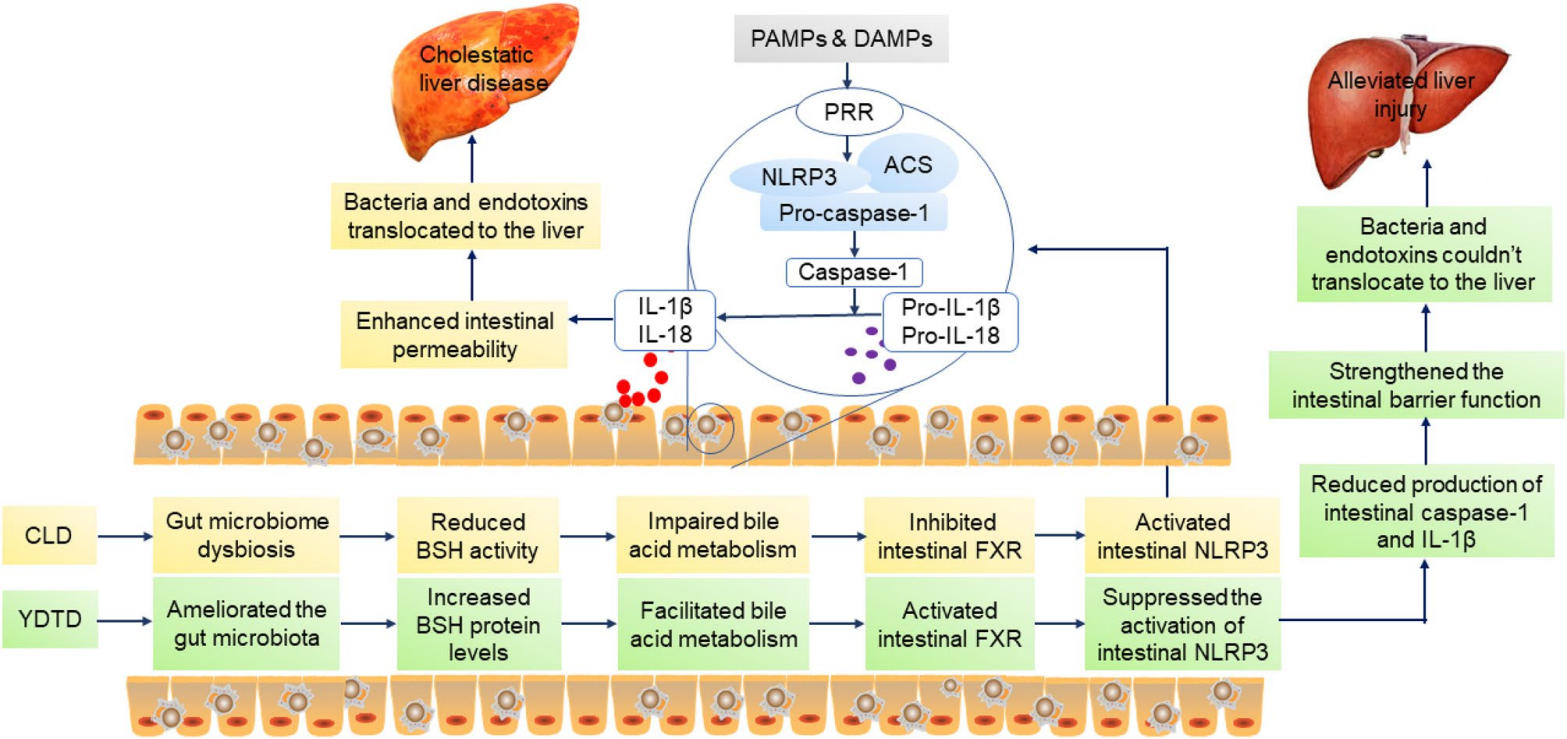
Schematic illustration of the proposed mechanism by which YDTD alleviates cholestatic liver injury. YDTD reshapes the gut microbiota, enriching BSH-producing bacteria (e.g., *Lactobacillus* and *Bifidobacterium*), which enhances bile acid deconjugation and promotes the conversion of primary bile acids to secondary bile acids. This leads to increased levels of unconjugated and secondary bile acids that activate intestinal FXR signaling. FXR activation subsequently suppresses NLRP3 inflammasome-mediated inflammation, restores intestinal barrier integrity (upregulating tight junction proteins such as occludin and ZO-1), and ultimately ameliorates cholestatic liver injury

Collectively, these results indicate that YDTD promotes bile acid deconjugation and biotransformation by modulating gut microbiota composition and enhancing BSH activity, as summarized in the proposed mechanistic model (Fig. 8).

## Discussion

This study demonstrates that YDTD alleviates liver injury by modulating the gut microbiota–bile acid–FXR–NLRP3 signaling axis. Specifically, YDTD enriched beneficial bacterial populations (e.g., *L. murinus* and *B. pseudolongum*), upregulated BSH protein expression, and enhanced the conversion of conjugated bile acids to unconjugated and secondary bile acids, which are more readily excreted. This process reduced pathological bile acid accumulation and alleviated cholestasis. In turn, unconjugated and secondary bile acids activated intestinal FXR signaling, inhibited the NLRP3 inflammasome, and strengthened the intestinal barrier, thereby limiting bacterial and endotoxin translocation to the liver and mitigating hepatic inflammation.

Previous studies have indicated that several key components of YDTD—such as *Artemisiae Scopariae Herba*, *Lysimachiae Herba*, *Indigo Naturalis*, *Poria*, and *Atractylodis Macrocephalae Rhizoma*—may modulate gut microbiota composition by inhibiting harmful bacteria and promoting beneficial microbial populations, thereby supporting intestinal barrier function and liver health [23–30]. Consistent with these findings, our analysis revealed that YDTD treatment in CLD mice selectively increased the proportion of beneficial bacteria, including *L. murinus* and *L.* johnsonii, which are known for their anti-inflammatory and barrier-strengthening effects [31, 32]. In addition, YDTD treatment increased the abundance of *B. pseudolongum* and *Bifidobacterium animalis*, species associated with improved mucosal defense and tight junction integrity [33, 34]. By contrast, CLD mice exhibited elevated levels of *A. muciniphila*, a mucin-degrading bacterium whose overgrowth can disrupt the mucus layer and promote inflammation under pathological conditions [35, 36]. YDTD treatment reversed this imbalance by reducing the abundance of *A. muciniphila* while restoring beneficial *Lactobacillus* and *Bifidobacterium* populations. These coordinated microbial shifts likely contribute to YDTD’s ability to attenuate intestinal inflammation, reinforce barrier function, and alleviate liver injury.

The gut microbiome plays a central role in regulating bile acid metabolism, which, in turn, modulates intestinal FXR signaling [22]. Key commensals such as *L. murinus* and *B. pseudolongum* are efficient producers of BSH, an enzyme that deconjugates bile acids into unconjugated forms [37, 38]. Unconjugated bile acids act as signaling molecules that influence metabolic processes [39] and are more readily excreted than conjugated bile acids [40]. Although conjugated bile acids (e.g., T-β-MCA, TUDCA, and TCDCA) antagonize intestinal FXR activity, unconjugated bile acids activate it. FXR serves as an important anti-inflammatory regulator of intestinal barrier function. Its expression is reduced in cholestasis, and its activation suppresses intestinal inflammation and barrier damage [41]. FXR also interacts directly with NLRP3 to inhibit inflammasome activation and downstream pro-inflammatory signaling [42]. In general, BSH rapidly hydrolyzes conjugated bile acids to alleviate FXR inhibition and promote its signaling [9, 42]. In this study, CLD mice exhibited gut dysbiosis with decreased abundance of beneficial bacteria and reduced intestinal BSH protein levels, impairing the hydrolysis of conjugated bile acids (e.g., T-α/β-MCA) and increasing the conjugated-to-unconjugated bile acid ratio. YDTD treatment increased the proportion of beneficial bacteria and BSH protein expression, enhanced conversion of bile acids to unconjugated and secondary bile acids, diversified the bile acid pool, and promoted fecal excretion of these bile acids. This restored bile acid profile likely improved intestinal barrier function via the FXR-NLRP3 pathway, reducing liver exposure to harmful gut-derived substances and supporting hepatic protection.

Collectively, our findings indicate that YDTD enriches *L. murinus* and *B. pseudolongum* in the gut and elevates intestinal BSH protein levels. These changes promote bile acid metabolism, activate intestinal FXR signaling, and inhibit NLRP3 inflammasome activation, ultimately improving intestinal barrier integrity and alleviating liver injury. Although these findings were established in an ANIT-induced acute cholestasis model, their translational relevance requires further validation in chronic models that better mimic human CLD—such as bile duct ligation, 3,5‑diethoxycarbonyl‑1,4‑dihydrocollidine diet, or Mdr2 knockout [43, 44]. Moreover, to definitively establish microbial causality, future studies should pursue: (1) isolation and in vitro BSH‑activity screening of bacterial strains from YDTD‑treated mice and (2) functional validation of the most active BSH‑producing strains using gnotobiotic animal models. Notably, our prior research identified bioactive YDTD components (e.g., liquiritigenin and L-arginine) with anti-inflammatory effects [14]. Future studies should functionally link these compounds to the gut microbiota-bile acid-FXR-NLRP3 axis, testing their modulation of BSH activity, FXR signaling, and inflammasome regulation. This integrated approach will unify the multi‑target immunomodulatory and microbiota‑directed mechanisms of YDTD. Although all conclusions drawn so far are derived from animal models, prospective clinical studies are essential to confirm the therapeutic relevance of this identified axis in humans. By conducting these follow‑up studies, we aim to prioritize experimental designs with sufficient sample sizes and appropriately powered cohorts to ensure statistical robustness and strengthen the reliability of our mechanistic conclusions.

## Data Availability

The datasets used and/or analyzed during the current study are available from the corresponding author on reasonable request.
